# Assessment of the plasmidome of an extremophilic microbial community from the Diamante Lake, Argentina

**DOI:** 10.1038/s41598-021-00753-1

**Published:** 2021-11-02

**Authors:** María Florencia Perez, Luis Alberto Saona, María Eugenia Farías, Anja Poehlein, Friedhelm Meinhardt, Rolf Daniel, Julián Rafael Dib

**Affiliations:** 1 Planta Piloto de Procesos Industriales Microbiológicos, Consejo Nacional de Investigaciones Científicas y Técnicas, San Miguel de Tucumán, Tucumán Argentina; 2 Genomic and Applied Microbiology and Göttingen Genomics Laboratory, Institute of Microbiology and Genetics, Georg-August University of Göttingen, Grisebachstr. 8, 37077 Göttingen, Germany; 3 Institut für Molekulare Mikrobiologie und Biotechnologie, Westfälische Wilhelms Universität Münster, Münster, Germany; 4 Instituto de Microbiología, Facultad de Bioquímica, Química y Farmacia, Universidad Nacional de Tucumán, San Miguel de Tucumán, Tucumán Argentina

**Keywords:** Microbiology, Archaea, Bacteria, Microbial communities, Environmental microbiology

## Abstract

Diamante Lake located at 4589 m.a.s.l. in the Andean Puna constitutes an extreme environment. It is exposed to multiple extreme conditions such as an unusually high concentration of arsenic (over 300 mg L^−1^) and low oxygen pressure. Microorganisms thriving in the lake display specific genotypes that facilitate survival, which include at least a multitude of plasmid-encoded resistance traits. Hence, the genetic information provided by the plasmids essentially contributes to understand adaptation to different stressors. Though plasmids from cultivable organisms have already been analyzed to the sequence level, the impact of the entire plasmid-borne genetic information on such microbial ecosystem is not known. This study aims at assessing the plasmidome from Diamante Lake, which facilitates the identification of potential hosts and prediction of gene functions as well as the ecological impact of mobile genetic elements. The deep-sequencing analysis revealed a large fraction of previously unknown DNA sequences of which the majority encoded putative proteins of unknown function. Remarkably, functions related to the oxidative stress response, DNA repair, as well as arsenic- and antibiotic resistances were annotated. Additionally, all necessary capacities related to plasmid replication, mobilization and maintenance were detected. Sequences characteristic for megaplasmids and other already known plasmid-associated genes were identified as well. The study highlights the potential of the deep-sequencing approach specifically targeting plasmid populations as it allows to evaluate the ecological impact of plasmids from (cultivable and non-cultivable) microorganisms, thereby contributing to the understanding of the distribution of resistance factors within an extremophilic microbial community.

## Introduction

Plasmids are self-replicating, extrachromosomal, mobile, genetic elements of ecological importance, as they may confer functions or beneficial traits enabling their hosts to thrive in a given environment and—equally important—they can act as horizontal gene transfer vehicles^1^ thereby contributing to the spread of genetic information within a microbial community. Thus, plasmids act as important evolutionary driving force by accelerating genome innovation and allowing acquisition of evolutionary novelty^2^.

There are myriads of studies addressing bacterial and archaeal plasmids, which eventually revealed the typical functions ensuring plasmid replication, mobilization and maintenance, as well as other accessory characteristics^3–12^. Only quite recently, however, it was recognized that to characterize them as a whole is necessary to understand their ecological impact in microbial ecosystems^13–18^. In this regard, the total plasmid DNA present in an ecological niche was defined as the “plasmidome”^19^. Indeed, plasmidome studies based on independent microbial culture methods substantiated the significance of extrachromosomal genetic elements with respect to different environments, such as the bovine rumen and wastewater treatment plants^13–15^. We have recently reported the study of the plasmidome of the Puquio de Campo Naranja, an extreme environment of the Puna Argentina^18^. However, despite the ecological relevance of plasmidomes such studies were only rarely performed, which is possibly due to the complexity of sample processing and the need of tailor-made bioinformatics tools for analysis.

**Figure 1:**
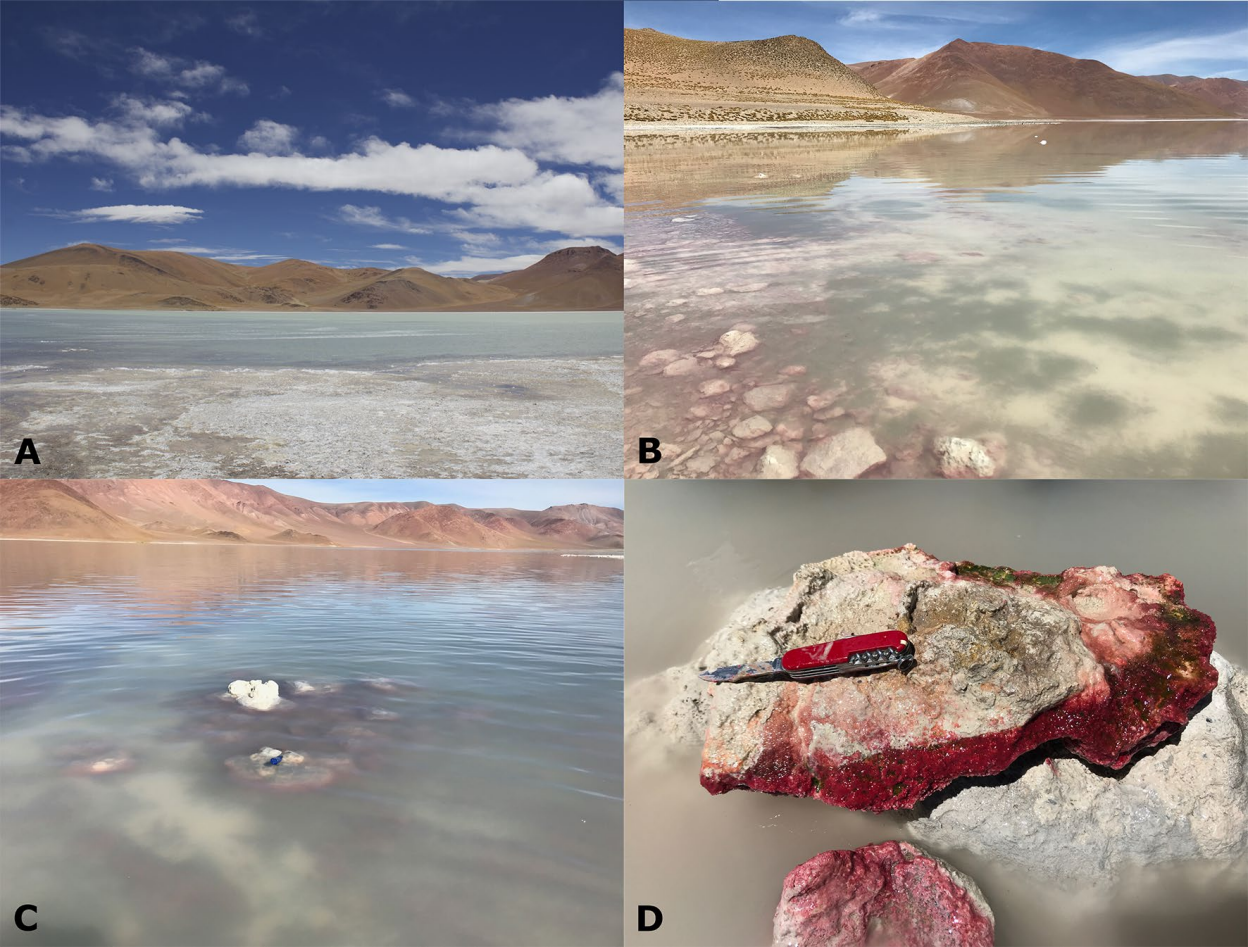
(**A**) Panoramic photography of the Diamante Lake, located in the Catamarca province, Argentina. (B-C) Submerged microbialites. (**D**) Red biofilms attached to gaylussite crystals at the bottom of the submerged microbialite.

Andean Puna constitutes a large reservoir of Andean Microbial Ecosystems (AMEs) including biofilms, microbial mats, microbialites and endoevaporites, all of which exposed to multiple extreme conditions such as high UV irradiation, and—due to the high altitude—low oxygen pressure, large thermal fluctuations, high dryness, hypersalinity, alkalinity, high concentrations of heavy metals and metalloids such as arsenic^20,21^. The highest concentration of As (up to 347 mg L^−1^ in the summertime) was reported for the Diamante Lake^22^, which is located inside the Galán Volcano boiler (40 km diameter) at an altitude of 4589 m above sea level in the Catamarca province, Argentina (Fig. 1). Physico-chemical parameters include high pH-values (9–11), elevated salinity (270 g L^−1^, 217 mS cm^−1^), strong UV irradiation (84 W m^−2^ of UV-AB at noon) and vast day–night temperature ranges (− 20 °C to + 20 °C)^23^. Microbial communities facing such an environment need to develop capacities ensuring survival. As stated above, extrachromosomal genetic elements could procure the genes enabling microbes to withstand harsh environmental conditions^24^. Rascovan et al.^25^ reported the discovery of a red biofilm consisting of 94% archaeal representatives, with members belonging to the class of the haloarchaea dominating. In the conventional metagenomic studies already carried out on such microbial communities^25,26^, a high abundance of genes related to anaerobic arsenate respiration (*arr*) as well as arsenite oxidation (*aio*) was detected, strongly supporting the assumption that arsenic is used in bioenergetic processes. In addition, genes related to detoxifying mechanisms for removal of intracellular arsenic were also found, e.g. the *acr3* gene and the *arsABCRD* operon. However, in those studies, the impact of plasmids is possibly underestimated because it is difficult to discriminate them from the chromosomal DNA, due to their low number of copies and corresponding low proportion. In this study, we assessed the plasmidome of the red biofilm from Diamante Lake (Fig. 1D), and compare it to the previously reported plasmidome of microbialites from Puquio de Campo Naranja^18^. In addition, we compared it to the plasmidome from a wastewater-treatment-plant containing effluents of the chemical/pharmaceutical industry (WWTP Visp, Switzerland) as it concerns an environment similarly loaded with high concentrations of metals^15^. Potential hosts as well as encoded functions were identified.

## Materials and methods

### Sampling and biomass purification

Samples were aseptically taken from red biofilms attached to gaylussite crystals at the bottom of submerged microbialites in the Diamante Lake, Catamarca, Argentina (26°00′51.04″S, 67°01′46.42″W) in September 2019 (Fig. 1). Microbialites were found at a distance of 2 m from the lake shore. Samples from three randomly chosen sites were taken and pooled to ensure representativeness. Stored in sterile plastic flasks at 4 °C such pooled samples were further processed within a week. Permission for sample collection was granted by the Secretaría de Medio Ambiente, Catamarca, Argentina (No. 22935/2016).

Microorganisms were separated from the sample by using the protocol described by Perez et al.^18^. Pellets obtained from biomass purification were kept at − 20 °C until plasmid DNA extraction.

### DNA extractions

The plasmid DNA was isolated by using the Large-Construct kit as recommended by the manufacturer (Qiagen, Hilden, Germany).

In parallel, metagenomic DNA was extracted from red biofilm samples by using the FastDNA Spin kit for soil as recommended by the manufacturer (MP Biomedicals, CA, USA). The extracted metagenomic DNA served as template for 16S rRNA gene amplicon sequencing.

### Chromosomal DNA removal

The plasmid DNA was subjected to overnight digestion at 37 °C with exonuclease V Rec BCD (New England Biolabs, Massachusetts, USA) to remove chromosomal DNA. PCR reactions using universal primers covering the V4 region of 16S rRNA gene were performed to check for chromosomal DNA contamination. The primers used for bacteria were F5′-CCTACGGGNGGCWGCAG-3′ (Bac_341F) and R5′-GGATTAGATACCCBDGTAGTC-3′ (Bac_785R)^27^; and for archaea F5′-CCCTAYGGGGYGCASCAG-3′ (Arc_340F) and R5′-ATTAGAKACCCSNGTAGTCC-3′ (Arc_806R)^28^. The plasmid DNA was purified using the SureClean Plus kit (Bioline, London, UK).

### Sequencing, quality control and assembly

Illumina shotgun paired-end sequencing libraries were generated from isolated plasmid DNA using the Nextera XT sample preparation kit as recommended by the manufacturer (Illumina, CA, USA). The MiSeq system together with the MiSeq reagent kit version 3 (600-cycle) was used for the plasmidome sequencing as recommended by the manufacturer (Illumina). The quality control of raw sequence reads was carried out with FastQC v0.11.9 and the reads were quality-filtered using Trimmomatic v0.38.0^29^. Finally, the reads were de novo assembled by using SPAdes software v3.9.0 with the -meta parameter to call the metaSPAdes module^30^. Recycler algorithm was used to assemble cyclic sequences, which are likely plasmids, phages and other circular elements from the assembly graphs provided by SPAdes^31^. The bioinformatic analysis pipeline described by Kothari et al.^17^ was also used to identify the complete closed circular contigs. The circular elements obtained in both cases were compared to DoriC 10, a database of replication origins in prokaryotic genomes including chromosomes and plasmids^32^.

### Bioinformatic analysis

The reads generated by sequencing of the plasmid DNA were aligned with the metagenome contigs using the Bowtie2 tool^33^. Metagenome contigs were assembled using SPAdes v3.9.0 from sequencing data of three independent red biofilm samples taken on another occasion and published by Saona et al.^26^.

Annotation and labeling of all the relevant genomic characteristics on plasmidome contigs were done with Prokka v1.14.5^34^. The assembled plasmidome dataset was submitted to the MG-RAST server^35^ for functional and taxonomic analysis. Comparisons with the SEED subsystem database were performed by using a maximum *E*-value of 10^–5^. The deduced functional profile of the red biofilm plasmidome was compared with the one derived from the metagenome mentioned above^26^ by employing the software STAMP (Statistical Analysis of Metagenomic Profiles)^36^. Comparisons with other plasmidomes were also performed^15,18^.

Both the known plasmid sequences from NCBI database and the domains related to plasmid replication and mobilization were assessed as described previously by Perez et al.^18^. In addition, the plasmidome contigs were compared to TADB 2.0 database by blastn in order to identify toxin–antitoxin (TA) systems^37^. Furthermore, the Prokka annotation file of the plasmidome was subjected to Conditional Reciprocal Best BLAST (*crb-blast*) against plasmid genes sequences from the ACLAME database with an *E*-value ≤ 10^–3^^38^. Hits with an identity ≥ 70% and an alignment coverage ≥ 90% were selected. Similarly, putative genes encoding metal resistance and virulence factors were also searched by using the BacMet^39^ and VFDB databases^40^, respectively.

Due to the high arsenic concentration found in the lake^22^, arsenic resistance-related genes were separately annotated. For this purpose, the amino acid sequences were downloaded from Uniprot and were subjected to Position-Specific Iterated BLAST (PSI-BLAST)^41^. CD-HIT v4.8.1 was used for creating non-redundant datasets^42^ and Clustal Omega v1.2.4 for sequence alignments^43^. Profiles Hidden Markov model were build and searched for in the plasmidome translated gene sequences identified with Prokka by using HMMER 3.3 (cut-off *E*-value < 10^–3^)^44^.

The Resistance Gene Identifier (RGI) software was employed for prediction of antibiotic-resistance genes using the Comprehensive Antibiotic Resistance database (CARD)^45^ as a reference.

The ISEScan software pipeline was used to search for mobile elements such as insertion sequences^46^, and the HMM profiles downloaded from TnpPred web^47^ for prediction of prokaryotic transposases by HMMER 3.3.

### Amplicon sequencing and taxonomic analysis

16S rRNA gene amplicon sequencing was performed using the above-described primers partially covering the 16S rRNA gene sequence. The MiSeq system together with MiSeq reagent kit version 3 (600-cycle) was used for sequencing of the amplicons as recommended by the manufacturer (Illumina). Data quality control and analysis were performed using the QIIME software^48^. First, paired-end reads were joined with PEAR v0.9.6^49^. Quality-filtering was performed using the split_libraries_fastq.py script. Forward and reverse primers were removed by using cutadapt v1.16^50^. USEARCH v11^51^ was used for zero-radius operational taxonomic unit (zOTU) determination. Taxonomy was assigned against Silva 132 database^52^.

## Results and discussion

### Sequencing and assembly output

Illumina sequencing from the Diamante Lake plasmidome generated 1,071,941 paired-end reads, of which 941,587 passed quality-filtering. The SPAdes assembler produced 13,492 contigs (> 500 bp) corresponding to roughly 16.9 Mb (largest contig 20.415 bp) (Supplementary Table S1). It is smaller than the previously reported one for another similar extremophile community of Puquio de Campo Naranja (135,813 contigs, 127.9 Mb)^18^.

Thirty-nine closed replicons were predicted by the Recycler software (> 1000 bp), the largest consisting of 3313 bp, but none displayed a known plasmid origin of replication when compared to the DoriC 10.0 database. The bioinformatic pipeline used to detect circularity produced 20 circular contigs. The largest comprised 8295 bp and the smallest 2025 bp. Only one of them showed a known plasmid origin of replication (87% similarity, alignment length of 70 nt), corresponding to pSN found in *Haloterrigena thermotolerans* strain H13 (DoriC ID: pORI00000477). Exclusively hypothetical proteins could be annotated from the open reading frames present in the circular elements.

### Functional analysis

MG-RAST analysis revealed that the Diamante Lake plasmidome contains a large fraction of unknown DNA as 18,314 sequences (41.5%) code for predicted proteins with known functions but 25,800 sequences (58.5%) encode putative proteins of unknown function. Thus, such deep-sequencing approaches are suited to detect novel proteins with so far undiscovered functions. In our previous plasmidome analysis from an AME, 39% of the predicted proteins could not be functionally annotated^18^. The difference between the two environments displaying rather similar environmental characteristics is possibly due to the large proportion of archaea present in the Diamante Lake, as archaeal genomes contain typically a higher fraction of “dark matter” when compared to bacterial genomes. The isolation and cultivation of most of the archaea, and accordingly, the experimental characterization of archaeal gene products, is challenging^53^. Likewise, Sentchilo et al.^15^ also reported 52 and 66% of coding sequences without assigned function in two wastewater treatment plant plasmidomes.

**Figure 2:**
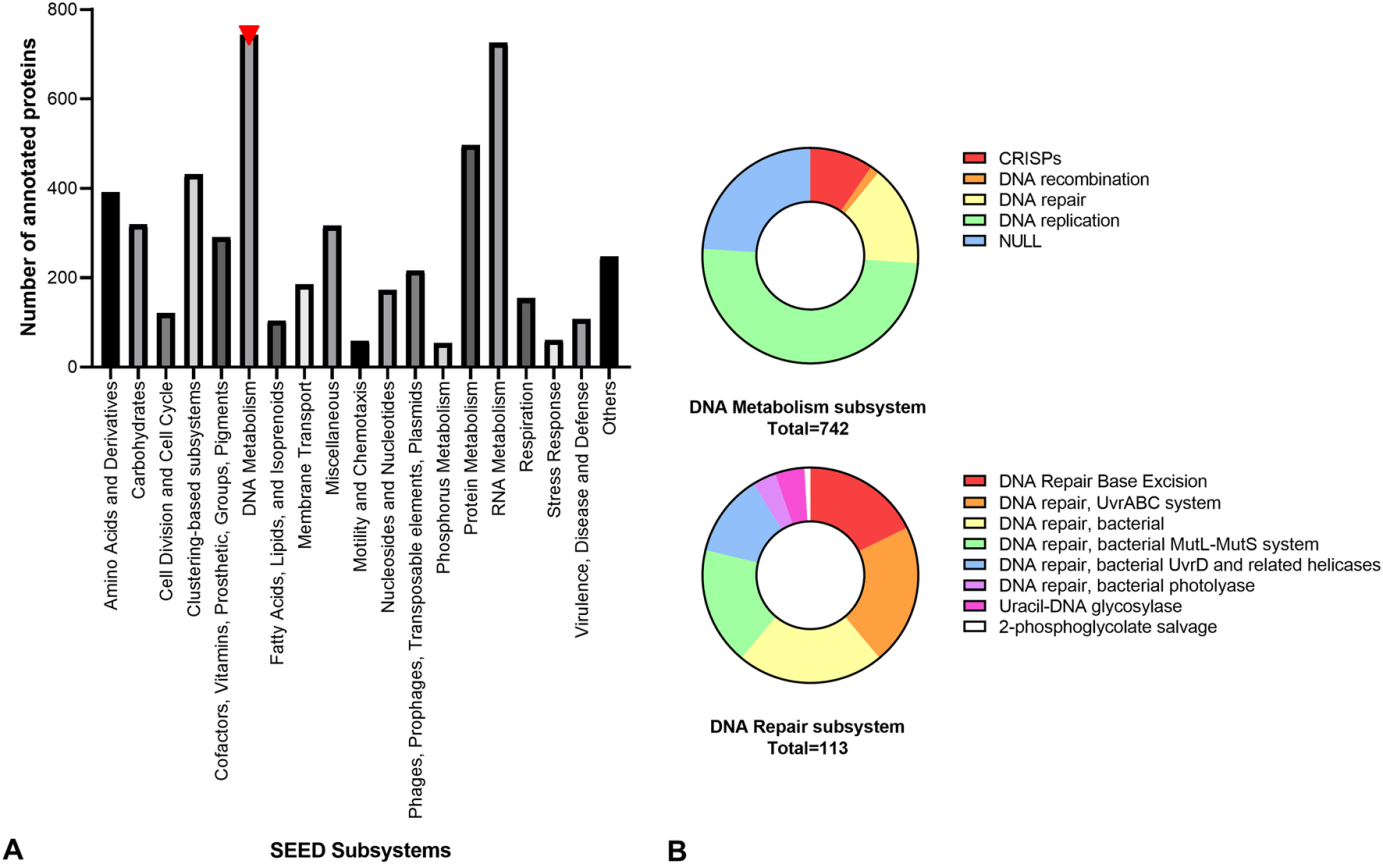
Predicted functional profile of the Diamante Lake plasmidome. (**A**) Number of annotated proteins of each of the major SEED subsystems by using a maximum *E*-value of 10^–5^. SEED subsystems with less than 50 hits were grouped into “Others”. (**B**) On top, “DNA Metabolism” subsystem level 2 classification of the SEED database. On the bottom, “DNA Repair” subsystem level 3 classification of the SEED database (*E*-value ≤ 10^–5^).

From the functional SEED assignment, only 5196 predicted proteins were annotated (28.4%), most of them covering basic metabolic functions such as DNA, RNA and protein metabolism (Fig. 2A). It is noteworthy that among the proteins involved in DNA metabolism those related to DNA repair were rather diverse (Fig. 2B). It is well known that DNA repair plays a key role as an adaptive mechanism to withstand the high UV irradiation in the Andean Puna^54–58^.

**Figure 3:**
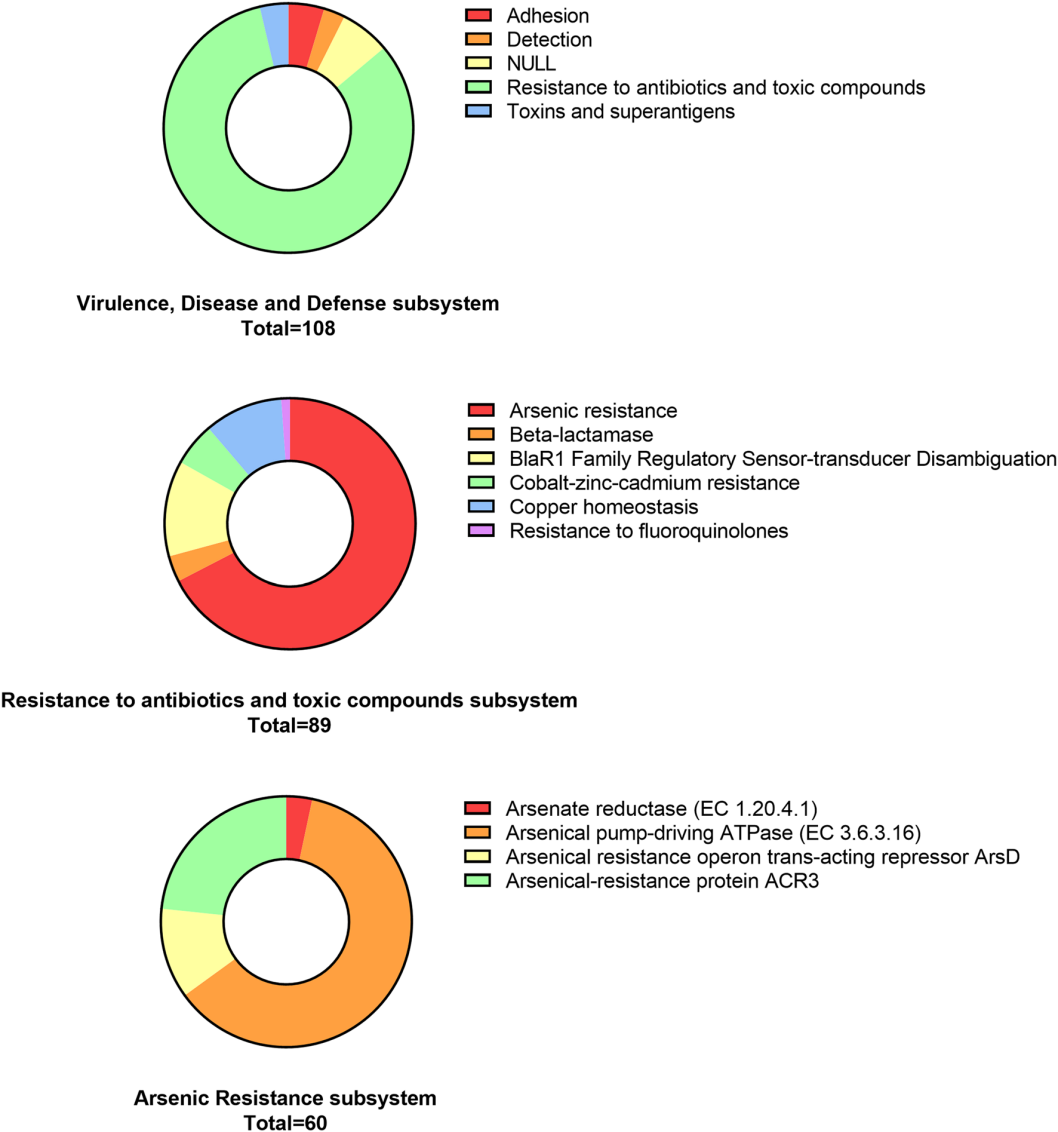
Predicted functional profile of the Diamante Lake plasmidome. On top, “Virulence, Disease and Defense” subsystem level 2 classification of the SEED database. In the middle, “Resistance to antibiotics and toxic compounds” subsystem level 3 classification of the SEED database. On the bottom, “Arsenic resistance” subsystem level 4 classification of the SEED database (*E*-value ≤ 10^–5^).

Although on a smaller scale, the subsystems "Stress Response" and "Virulence, Disease and Defense" were represented (Fig. 2A). With respect to stress response, predicted proteins involved in the response to oxidative stress were most frequently found (57.4%) (Supplementary Fig. S1). As for the DNA repair mechanisms, oxidative stress response systems contribute to protect microorganisms from UV-mediated damage. 82.4% of the assignments to the above mentioned second group corresponded to the subsystem “Resistance to antibiotics and toxic compounds”, with arsenic resistance systems prevailing (67.4%). The arsenic resistance included predicted proteins for an arsenate reductase (ArsC), an arsenical pump-driving ATPase (ArsA), an arsenical resistance operon trans-acting repressor (ArsD) and an arsenical-resistance protein ACR3 (Fig. 3).

The unique characteristics of this environment, such as its location at a high altitude, the exposure to extreme conditions and the peculiarities of its microbial composition, which includes a major proportion of archaea, may explain the relatively few functional annotations during grouping into SEED categories.

### Functional comparison between plasmidomes

**Figure 4:**
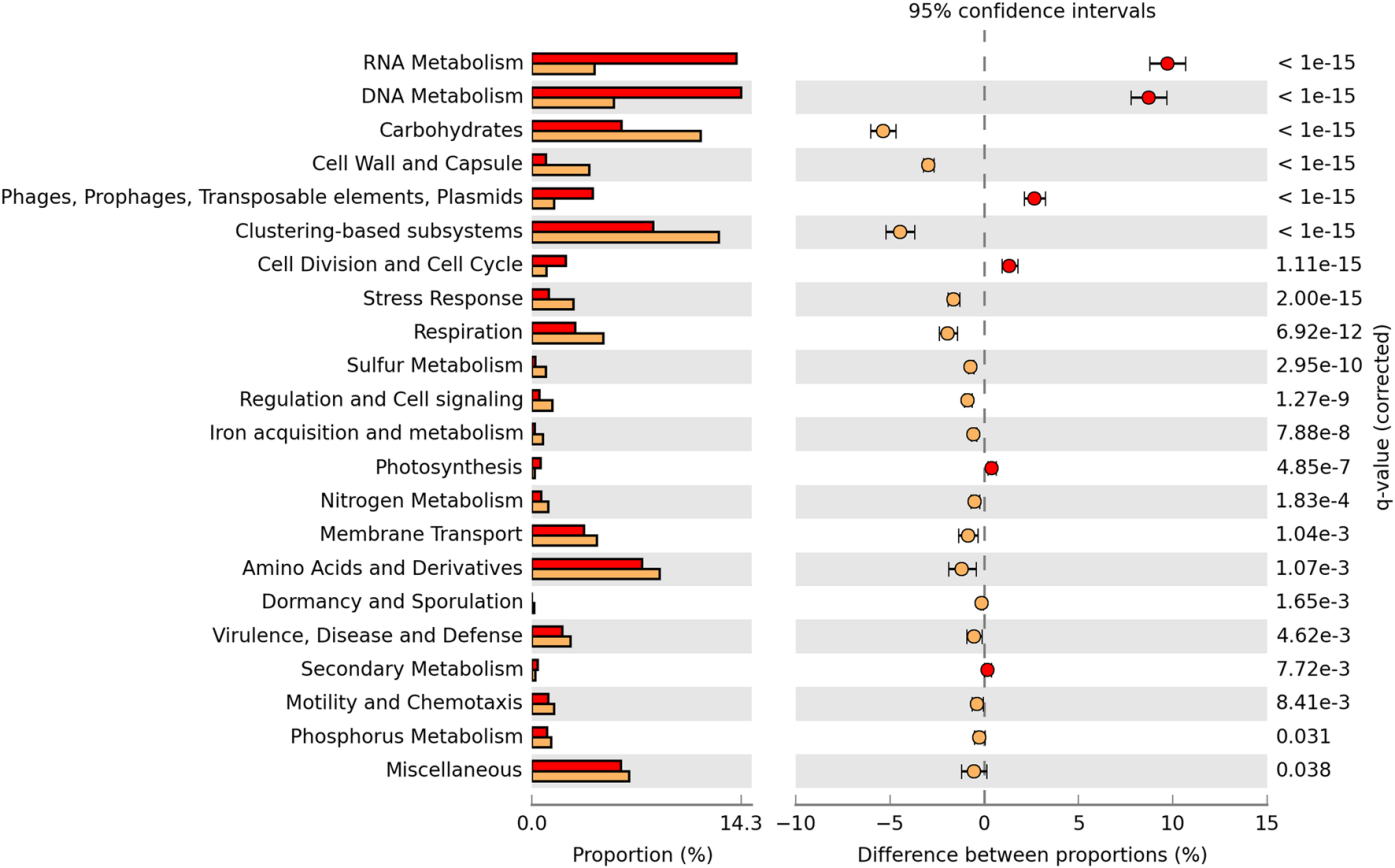
Predicted functional profiles derived from the Diamante Lake plasmidome (red bars) and the Puquio de Campo Naranja plasmidome (orange bars). Percent SEED categorizable protein-encoding genes and pairwise proportional differences calculated using STAMP. Fisher’s exact test was used and corrected P-values were calculated using Storey’s FDR. Only the statistically significant SEED subsystems are shown (q < 0.05).

The predicted functional profile of the Diamante Lake plasmidome was compared to the one derived from the Puquio de Campo Naranja plasmidome^18^. “RNA Metabolism”, “DNA Metabolism”, “Phages, Prophages, Transposable elements, Plasmids” and “Cell Division and Cell Cycle” subsystems were more frequently represented in Diamante Lake than in the other, while “Carbohydrates”, “Cell Wall and Capsule”, “Clustering-based subsystems”, “Stress Response”, “Respiration” were more abundant in Puquio de Campo Naranja. No significant differences in the abundances of other subsystems were observed, suggesting a certain degree of similarity of the predicted functional profiles for both of the AMEs (Fig. 4).

**Figure 5:**
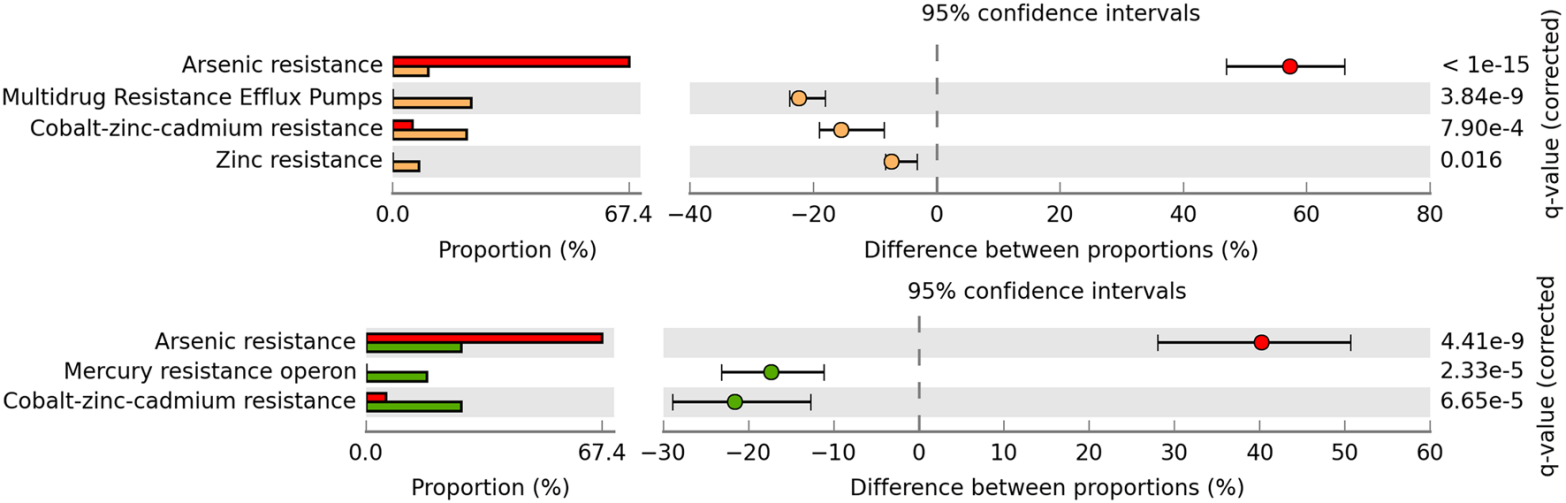
Predicted functional profiles derived from the Diamante Lake plasmidome (red bars), the Puquio de Campo Naranja plasmidome (orange bars) and the one derived from the wastewater treatment plant plasmidome in Visp, Switzerland (green bars). Percent “Resistance to antibiotics and toxic compounds” categorizable protein coding genes belonging to the “Virulence, Disease and Defense” SEED subsystem, and pairwise proportional differences calculated using STAMP. Fisher’s exact test was used and corrected P-values were calculated using Benjamini–Hochberg’s FDR. Only the statistically significant SEED subsystems are shown (q < 0.05).

Diamante Lake is known to be among the aqueous environments displaying high arsenic concentrations^22,25,59^, reinforcing the repeatedly mentioned presence of genes related to its resistance. Proportional differences between “Resistance to antibiotics and toxic compounds” categorizable protein coding genes belonging to the “Virulence, Disease and Defense” SEED subsystem showed that arsenic resistance is more abundant in the Diamante Lake plasmidome than in that of the Puquio de Campo Naranja (Fig. 5). In addition, the comparison of the former with that of a wastewater treatment plant containing effluents from chemical/pharmaceutical industries (WWTP Visp, Switzerland)^15^ revealed that arsenic resistance traits were again more abundant in the Diamante Lake plasmidome (Fig. 5).

### Plasmid-purification advantage

Plasmids usually represent only a small fraction of the total DNA in a given environment, due to their low rate of occurrence and number of copies. Hence, though they are casually recorded by conventional metagenomic sequencing methods, experimental plasmid-purification prior to sequencing allows for an analysis specifically targeting plasmid populations in a culture-independent manner, at best without losing information^60^. Obtained results are in line with such notion, as only 52% of plasmidome reads aligned with metagenome contigs described above in Saona et al.^26^. The same applies to the Puquio de Campo Naranja plasmidome, in which alignment reached only 30%^18^. Thus, our study strengthens that plasmid-purification prior to sequencing more satisfactorily meets the requirements to comprehensively assess the ecological importance of plasmid-borne sequences.

**Figure 6:**
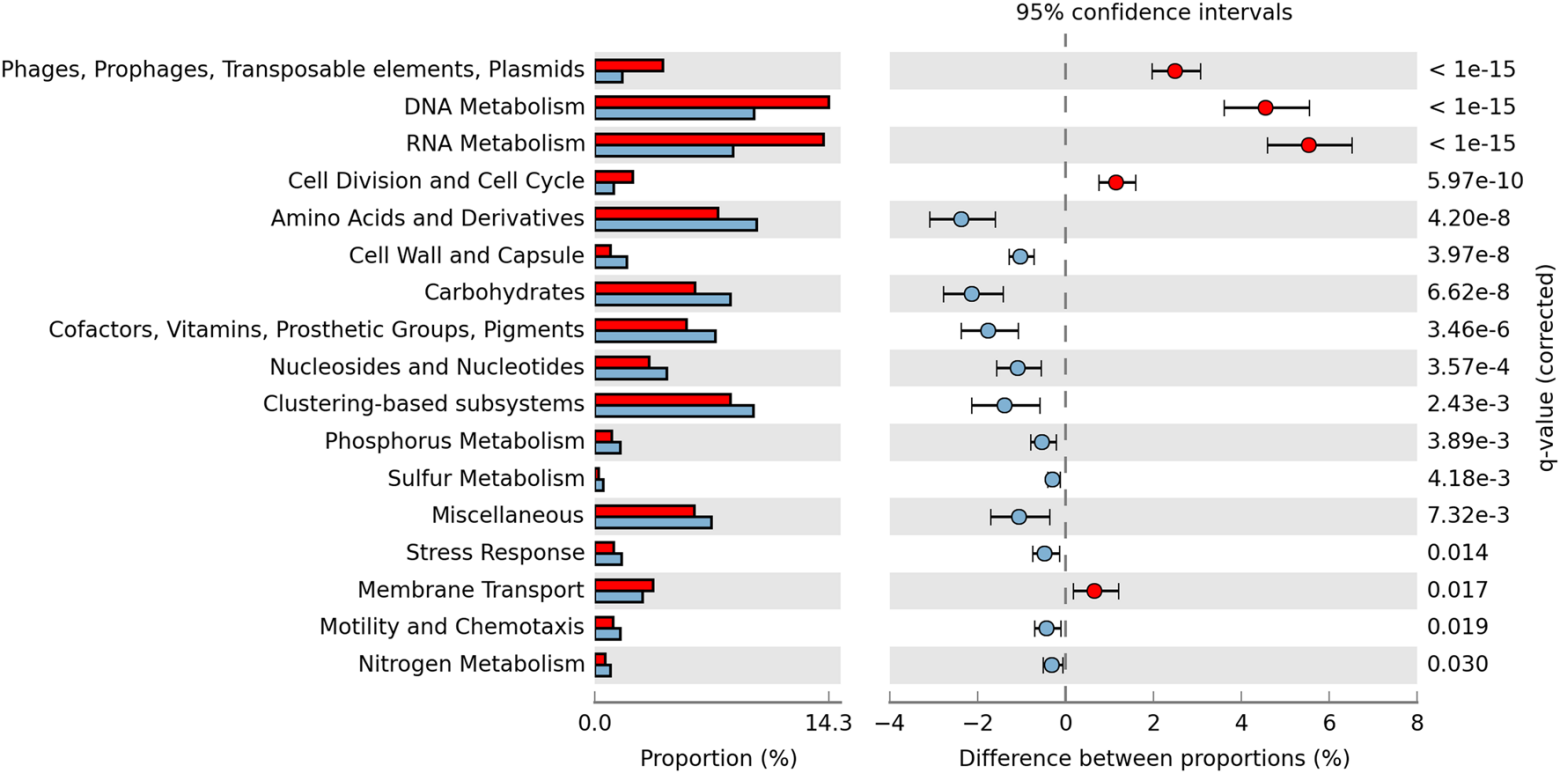
Predicted functional profiles derived from the plasmidome (red bars) and the metagenome (blue bars) of Diamante Lake red biofilm. Percent SEED categorizable protein-encoding genes and pairwise proportional differences calculated using STAMP. G-test (w/Yates’) was used and corrected P-values were calculated using Benjamini–Hochberg’s FDR. Only the statistically significant SEED subsystems are shown (q < 0.05).

The pairwise comparison aiming at distinguishing the plasmid gene pool from the metagenomic one (Fig. 6) accordingly revealed that “Phages, Prophages, Transposable elements, Plasmids” and “Membrane Transport” subsystems are more frequently represented in the plasmidome.

### Plasmid backbone functions: replication, mobilization and maintenance

In order to identify plasmid-like traits within the plasmidome, we focused on the search for Pfam domains related to plasmid replication and MOB-type relaxase families, which are related to plasmid mobilization.

RHH_1 and DUF1424 were detected as the main Pfam domains of plasmid replication in the Diamante Lake plasmidome, followed by RepL (Table 1). Likewise, RHH_1 and RepL protein families were also the most abundant in the plasmidome from Puquio de Campo Naranja^18^. It was not the case of DUF1424, which is a family of several archaeal proteins that seems to be present exclusively in *Halobacterium* and *Haloferax* species. Although the function of the latter family is unknown, its members are probably rep proteins due to the presence of conserved functional motifs^61,62^.

**Table 1:** Plasmid replication-related Pfam in the Diamante Lake plasmidome.

Pfam name	Pfam code	Description	Hits
Rep_1	PF01446.17	Replication protein	45
Rep_2	PF01719.17	Plasmid replication protein	1
Rep_3	PF01051.21	Initiator Replication protein	5
RepL	PF05732.11	Firmicute plasmid replication protein (RepL)	85
TrfA	PF07042.11	TrfA protein	3
RepA_C	PF04796.12	Plasmid encoded RepA protein	2
Rep_trans	PF02486.19	Replication initiation factor	0
RHH_1	PF01402.21	Ribbon-helix-helix protein, copG family	344
Rop	PF01815.16	Rop protein	0
RP-C	PF03428.13	Replication protein C N-terminal domain	32
RPA	PF10134.9	Replication initiator protein A	2
RepA_N	PF06970.11	Replication initiator protein A (RepA) N-terminus	5
RepC	PF06504.11	Replication protein C (RepC)	4
Replicase	PF03090.17	Replicase family	5
IncFII_repA	PF02387.15	IncFII RepA protein family	0
PriCT_1	PF08708.11	Primase C terminal 1 (PriCT-1)	4
DUF1424	PF07232.11	Putative rep protein (DUF1424)	179
Phage_CRI	PF05144.14	Phage replication protein CRI	0
PriCT_2	PF08707.11	Primase C terminal 2 (PriCT-2)	1
Prim-Pol	PF09250.11	Bifunctional DNA primase/polymerase, N-terminal	12
SSB	PF00436.25	Single-strand binding protein family	0
RepA1_leader	PF08048.12	Tap RepA1 leader peptide	0
DUF1738	PF08401.11	Domain of unknown function (DUF1738)	0
UvrD-helicase	PF00580.21	UvrD/REP helicase N-terminal domain	12
RepB-RCR_reg	PF10723.9	Replication regulatory protein RepB	30
Replic_Relax	PF13814.6	Replication-relaxation	73
KorB_C	PF06613.11	KorB C-terminal beta-barrel domain	0
KorB	PF08535.10	KorB domain	6
Activator-TraM	PF11657.8	Transcriptional activator TraM	1
pRN1_helical	PF13010.6	Primase helical domain	0
KORA	PF16509.5	TrfB plasmid transcriptional repressor	17
Rol_Rep_N	PF18106.1	Rolling Circle replication initiation protein N-terminal domain	0

Rep_1 and Rep_3 are the major families of replication initiation proteins. They have been reported among the most abundant in plasmidomes from wastewater treatment plants and a rat cecum^15,16,63,64^. In this study, domains belonging to the Rep_1 and Rep_3 families were also detected, but with a lower hit rate. Domains of replication initiation proteins from other known families were not detected (Table 1). Possibly, there are replication systems for which the molecular details and the mechanisms are currently unknown, particularly as most of the contributing microorganisms were not cultured and because of the taxonomic composition dominated by specific taxa, i.e. halobacteria^25^.

The most abundant relaxase families in the plasmidome were MOB_C_ and MOB_P_, for which 41 and 18 protein domain matches, respectively, were counted. MOB_T_, MOB_V_ and MOB_M_ were also present (3, 2 and 1 protein domain matches, respectively) (Table 2). Mobilization elements have been reported in most of the previous plasmidome analyses^14–16,63^, however, the classification in relaxase MOB families proposed by Garcillán-Barcia et al.^65,66^ was not performed. Meanwhile, 29 protein domain matches were counted for MOB_T_ family in the Puquio de Campo Naranja plasmidome^18^, and Kothari et al.^17^ reported the MOB_Q_ and MOB_P_ families as the most abundant in circular plasmids from groundwater plasmidomes.

**Table 2:** Relaxase MOB families in the Diamante Lake plasmidome.

Relaxase MOB family	Profile HMM	Hits
MOB_B_	T4SS_MOBB	0
MOB_C_	T4SS_MOBC	41
MOB_F_	Profile_MOBF	0
MOB_H_	T4SS_MOBH	0
MOB_P_	T4SS_MOBP1	18
	T4SS_MOBP2	0
	T4SS_MOBP3	0
MOB_Q_	T4SS_MOBQ	0
MOB_T_	Profile_MOBT	3
MOB_V_	T4SS_MOBV	2
MOB_M_	Profile_MOBM	1

In addition to the above plasmid replication and mobilization entries, sequences harboring genes involved in plasmid maintenance such as loci corresponding to toxin–antitoxin (TA) systems were identified (identity and coverage at least 85%). All of the TA systems belong to type II TA-loci (Supplementary Table S2). Only a single complete system could be annotated, i.e. a toxin with the respective antitoxin (T2787-AT2787), both located in the same contig (NODE_2880). It corresponds to the VapBC family, where the toxin is a PIN-domain ribonuclease (145 aa) and the antitoxin is a transcription factor (98 aa)^67^. Interestingly, this system is known from *Haloquadratum walsbyi* DSM 16790, a halophilic archaeon that was isolated from a solar saltern in Brac del Port (Alicante, Spain), and it was found to dominate most of the thalassic NaCl-saturated environments^68^.

In the previous plasmidome studies, TA systems were not taken into consideration. Only Kothari et al.^17^ reported the YoeB-YefM and RelE/StbE-RelB/StbD type II TA systems in some circular plasmids from groundwater plasmidomes. YoeB and RelE are ribosome-dependent RNase toxins that bind directly to the A site of the ribosome, where they cleave ribosome-associated mRNA^69^.

### Plasmid accessory functions: antibiotic resistance and arsenic resistance

Sequence analysis of the plasmidome from the Puquio de Campo Naranja revealed that antibiotic resistance traits are widespread in this extreme pristine environment, as 123 putative antibiotic resistance genes (ARGs) were annotated^18^. In the present study, only 8 ARGs could be classified, conveying resistance to 10 drug classes, among them macrolides, carbapenems, cephalosporins, penams (Supplementary Table S3). Such noticeable difference with respect to the number of ARGs found in similar extreme environments is probably due to microbial regional distinctions. The metabolic processes and the cell walls of bacteria and archaea display significant differences, offering an explanation for the fact that a number of antibiotics are effective against the former but do not threaten the latter^70^. Moreover, studying antibiotic-resistance mechanisms is—due to the clinical relevance—of much more necessity in the bacterial domain, as pathogenic archaea have not yet been identified^71^. So far, only a relationship between the periodontal-disease-severity and the relative abundance of the archaeon *Methanobrevibacter oralis* was reported^72^.

The bias necessarily introduced by the existing databases and developed from the information currently available, as well as the dominance of the archaea in the microbial community studied interfere with the analysis of the resistome encoded by the Diamante Lake plasmidome. Thus, it cannot to be excluded that the lack of relevant knowledge is the reason for the low number of identifiable ARGs and virulence factors.

Regarding the resistances to metals, a respective search in the BacMet database produced no hits, possibly also due the above reason as the database consists solely of bacterial entries. However, our manual annotation disclosed the presence of arsenic resistance genes. Arsenic hits various microorganisms, however, several bacteria and archaea possess detoxification systems enabling growth even under high As-concentrations^73^. The most common resistance system is encoded by the *ars* operon for which different genetic organizations were described among prokaryotes^74^. In addition to the extrusion systems, composed of the gene-products encoded by *arsA*, *arsB*, *arsC*, *arsD*, *arsR,* and *acr3*, another mechanism involving a putative arsenite(III)-methyltransferase (ArsM) was reported in *Halobacterium* sp. NRC-1^75^. We identified 28 proteins possibly related to arsenic resistance; ten of them had been automatically annotated as "hypothetical proteins" by Prokka (Supplementary Table S4). Hence, automatic annotation bears the risk of less accurate results or the disclosure of a fewer number of genes than actually exist. It is to be emphasized that genes enabling microorganisms to conduct anaerobic arsenate respiration (*arr*) and arsenite oxidation (*aio*) were not detectable in the plasmidome, as these are usually encoded by the chromosome.

As already mentioned, the genetic organization of the *ars* operons can vary among diverse microorganisms. In the Diamante Lake plasmidome, *arsC* (arsenate reductase) and *acr3* (arsenite efflux transporter) genes were present twice in close proximity, but the most relevant was the *arsADR* gene cluster of contig 116 (Supplementary Table S4). The genetic arrangement agrees with that described for pHLAC01 and pNRC100 of *Halorubrum lacusprofundi* ATCC 49239 and *Halobacterium* sp. NRC-1, respectively (Supplementary Fig. S2). In all cases, the *arsDA* and *arsR* genes are transcribed in opposite directions and the absence of the arsenite transporter ArsB-encoding gene is noticeable. This otherwise unusual operon structure is apparently characteristic for the haloarchaea^74,75^.

### Plasmid databases: NCBI and ACLAME

Sequences belonging to 24 megaplasmids described in 13 strains of halophilic archaea isolated from different saline environments were found (Table 3). Most of the matches were with the plasmid of *Halobacterium* sp. DL1 (315 kb), which was isolated from a freshwater pond (NZ_CP007061.1). Fourteen matches were detected between the plasmidome sequences and the sequences of the plasmid pHLAC01 (431 kb) of *Halorubrum lacusprofundi* ATCC 49239 that was isolated from the Deep Lake, a hypersaline Antarctic site. It is noteworthy that one of these matches (identity 92%, length 4123 bp) corresponds to contig 116 of the plasmidome, which harbors the *arsDA* genes (Supplementary Fig. S3). Thirteen sequences match with plasmid pHTIA (330 kb) of *Halorhabdus tiamatea* SARL4B, which was isolated from the Shaban deep-sea hypersaline anoxic lake in the Red Sea^76^. Thus, such plasmid sequences are evidently preserved in different high salinity environments.

**Table 3:** Plasmid matches of the Diamante Lake plasmidome with entries derived from the NCBI database.

Plasmid	Host	Size (kb)	Isolation source	Matches	NCBI accession number	References
pNRC100	*Halobacterium* sp. NRC-1	191	NE*	6	NC_001869.1	Ng et al. (2000)^82^
pNRC200		365		8	NC_002608.1	
pNG500	*Haloarcula marismortui* ATCC 43,049	132	Dead Sea	1	NC_006393.1	Baliga et al. (2004)^83^
pNG600		155		7	NC_006394.1	
PHS1	*Halobacterium salinarum* R1	147	NE	3	NC_010366.1	Pfeiffer et al. (2008)^84^
PHS4		41		1	NC_010367.1	
PHS3		284		4	NC_010368.1	
PHS2		195		2	NC_010369.1	
pHLAC01	*Halorubrum lacusprofundi* ATCC 49,239	431	Deep Lake, Antarctica	14	NC_012030.1	NA**
pHTUR01	*Haloterrigena turkmenica* DSM 5511	698	Saline soil in Turkmenistan	1	NC_013744.1	Saunders et al. (2010)^85^
pHV3	*Haloferax volcanii* DS2	438	Bottom sediment of the Dead Sea	3	NC_013964.1	Hartman et al. (2010)^86^
pHV4		635		4	NC_013966.1	
pHV1		85		1	NC_013968.1	
p1	*Halalkalicoccus jeotgali* B3	406	Salt-fermented seafood from South Korea	2	NC_014298.1	Roh et al. (2010)^87^
p2		363		1	NC_014299.1	
pHBOR02	*Halogeometricum borinquense* DSM 11,551	339	Solar salterns of Cabo Rojo, Puerto Rico	1	NC_014731.1	Malfatti et al. (2009)^88^
pHBOR01		362		1	NC_014735.1	
pHBOR03		210		1	NC_014736.1	
pHALXA01	*Halopiger xanaduensis* SH-6	436	Saline sediment of Lake Shangmatala, China	2	NC_015658.1	Anderson et al. (2012)^89^
pHALXA02		181		1	NC_015667.1	
pHTIA	*Halorhabdus tiamatea* SARL4B	330	Shaban deep-sea hypersaline anoxic lake in the Red Sea	13	NC_021913.1	Werner et al. (2014)^76^
pHH126	*Haloarcula hispanica* N601	125	Solar saltern in Alicante, Spain	1	NC_023011.1	Ding et al. (2014)^90^
pXH-48	*Halostagnicola larsenii* XH-48	131	Saline sediment of Lake Xilinhot, China	1	NZ_CP007057.1	Castillo et al. (2006)^91^
NA	*Halobacterium* sp. DL1	315	Fresh water, Ponds	23	NZ_CP007061.1	NA

*NE not specified, **NA not available.

When the red biofilm plasmidome genes annotated by Prokka were compared with plasmid genes of the Aclame database, 125 matches corresponded to genes of 11 megaplasmids of five different halophilic archaeal and one actinobacterial strain (*Rhodococcus* sp. RHA1) (Supplementary Table S5). Most of them were related to DNA metabolism, transposition and recombination. Gene related to arsenic resistance, DNA repair and plasmid partitioning were also identified. Thus, for many of the hypothetical proteins identified by Prokka a function was attributed, but not to all of them. Anyway, the comparison provided further justification of the practice as the presence of known plasmid-associated genes in our plasmidome dataset was proven.

### Mobile genetic elements: transposases and insertion sequences

A total of 532 insertion sequences (IS) were identified in the Diamante Lake plasmidome, with IS200/IS605 and IS5 like elements being the most frequent ones followed by members of the IS4, IS6, IS630 and ISH3 families (Supplementary Table S6). The first are the five main IS families spreading in Halobacteria, which is the dominating class of the studied community^77^. Most of the archaeal IS fall into families detected in Bacteria, while others are restricted to Archaea such as members of the ISH3 family^78^. Two new potential IS not attributable to any of the known families were classified as well (GenBank accession OK172335 and OK172336). In the Puquio de Campo Naranja plasmidome, a much lower number of IS (28) was reported. Again, most of them were assigned to the IS5, IS630 and IS4 families^18^. The absence of Tn3 family transposases in both of the plasmidomes is conspicuous, as it represents one of the most abundant families in bacterial genomes, and Tn3 elements preferentially transpose into plasmids^79^.

The presence of so many IS elements is in line with the notion that the plasmidome substantially contributes to genome evolution as well as adaptation processes by facilitating the acquisition of novel genes and beneficial traits^80,81^.

### Taxonomic analysis

Although plasmids can be transferred between different microorganisms, the taxonomic assignment of the plasmidome contigs allows an estimation of potential hosts. Eighty-eight percent were assigned to Archaea, while 11% were assigned to Bacteria and the remaining 1% to Eukarya. Among the Archaea, the phylum Euryarchaeota (99.85%) is dominating.

**Figure 7:**
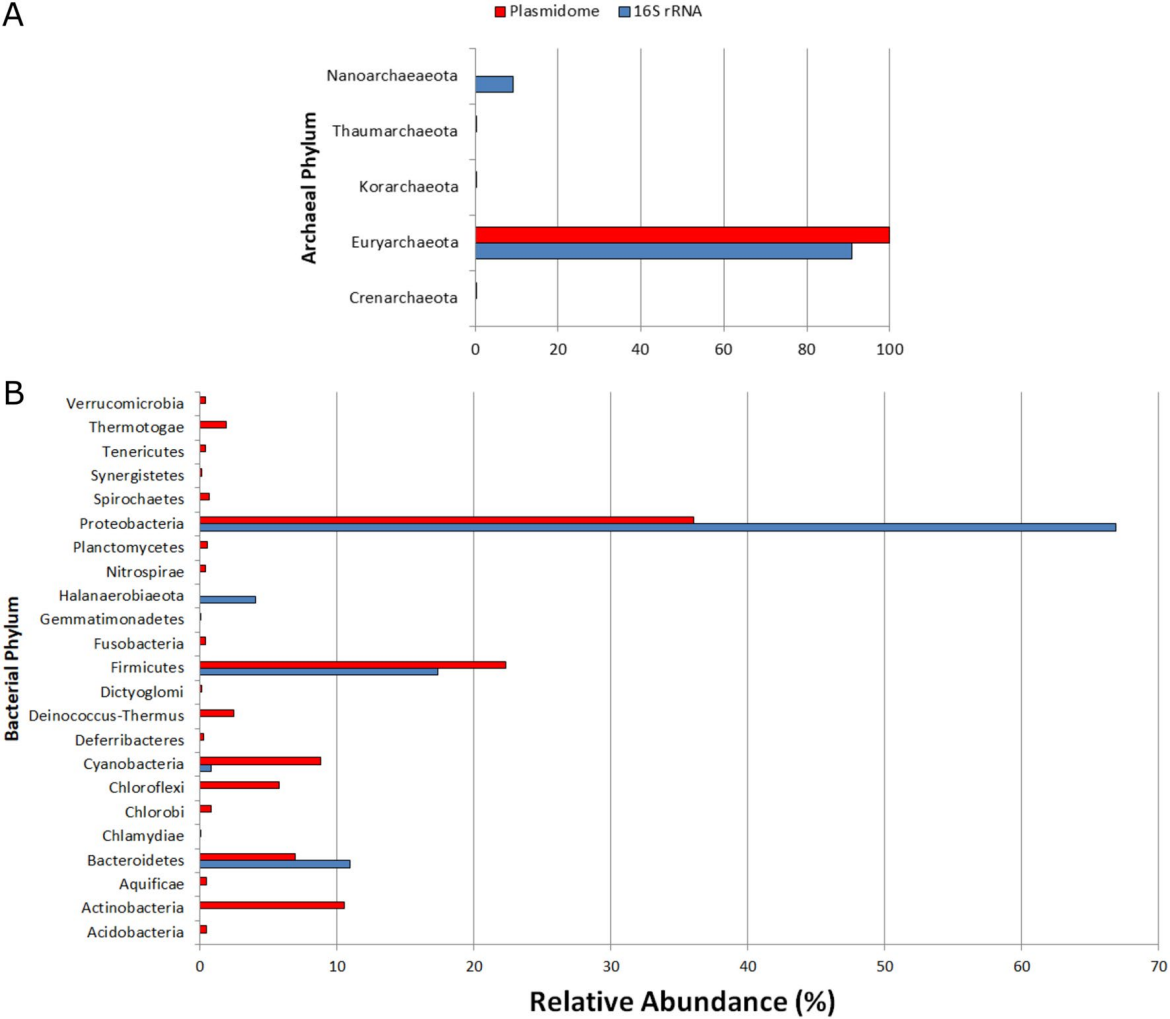
Red biofilm taxonomic analysis from Diamante Lake. Red bars show the relative abundance of each archaeal (**A**) or bacterial (**B**) phylum in the plasmidome by MG-RAST analysis using similarity to the RefSeq database (*E*-value ≤ 10^–5^). Blue bars show the relative abundance of each archaeal (**A**) or bacterial (**B**) phylum by metagenomic DNA analysis using 16S rRNA gene amplicon sequencing. Phyla with abundance less than 1% in both datasets were not included.

When the phylum distribution of the plasmidome was compared with 16S rRNA sequencing data from the corresponding metagenomic DNA sample, the phylum Euryarchaeota again stood out with the highest relative zOTU abundance (Fig. 7A). In both, the class of the halobacteria dominated. With respect to the Bacteria, the Proteobacteria (36%), the Firmicutes (22.3%) and the Actinobacteria (10.5%) comprised most of the contigs assigned in the plasmidome. Also, in the metagenome, the Proteobacteria (66.9%) and the Firmicutes (17.3%) account for the phyla with the highest relative zOTU abundance with the Bacteroidetes, however, ranking third (10.9%) (Fig. 7B). Notably, plasmid contigs of Actinobacteria, Chloroflexi and Deinococcus-Thermus were obtained, whereas the 16S rRNA analysis did not disclose members of these phyla. A possible explanation might be horizontal transfer of plasmid-borne genes between bacterial phyla, or the existence of plasmids with a wide host range. On the other hand, the 16S rRNA analysis indicates the presence of members of the phylum Halanaerobiaeota but no plasmidome contig could be assigned to the latter, which is possibly due to a bias in plasmid databases when a phylum is not well represented or to the absence of plasmids within the taxon.

## Conclusions

It is currently possible to study plasmid elements in the course of a conventional metagenomic analysis, but an approach to specifically target plasmid populations allows to overcome the inherent constraints of the bioinformatic tools applied for the analysis of plasmids from total community DNA. Under this perspective, and from the comparison with the metagenome of the same community, this study showed that part of the plasmid information will not be detected when the experimental plasmid-purification is not carried out prior to sequencing. Furthermore, a large fraction of genes with an unknown function was present in the plasmidome dataset, as at least 58.5% of the predicted proteins were hypothetical. In addition, the percentages of SEED assignments were even lower. The relatively few functional annotations may accord with the peculiarities of the extreme environment, which harbors a microbial community that is dominated by archaea. On the other hand, functions related to the response to oxidative stress and DNA repair were annotated, which agrees with the requirement of adaptive mechanisms enabling the hosts to withstand the exposure to the high UV irradiation in the Andean Puna.

Comparison of the Diamante Lake plasmidome to that of Puquio de Campo Naranja, revealed a certain degree of similarity between the predicted functional profiles of both AMEs. However, striking differences with respect to antibiotic and arsenic resistance were detected. Sequences pointing to arsenic resistance are more abundant in the Diamante Lake plasmidome, a fact that also accounts for the plasmidome derived from a wastewater treatment plant that contains large quantities of effluents of the chemical/pharmaceutical industry. Our results reflect the high amount of arsenic present in the environment under investigation. Traits expected to be found in a plasmid pool were detected, such as Pfam domains related to plasmid replication, MOB-type relaxase families related to plasmid mobilization and genes belonging type II toxin–antitoxin systems related to plasmid maintenance. Moreover, there are sequences known from megaplasmids of halophilic archaea isolated from different saline environments, which provides further evidence for known plasmid-associated genes in the obtained dataset.

The results presented here along with the detection of numerous IS elements favors the opinion that the plasmidome facilitates the mobility and the transfer of genes within such extreme microbial communities.

## Supplementary Information

Supplementary Figures.

Supplementary Tables.

## Data Availability

The sequence data of the Diamante Lake plasmidome and 16S rRNA gene amplicon have been deposited at NCBI (National Center for Biotechnology Information) under the accession numbers SRR13795604, SRR13795605 and SRR13795606.

## References

[1] Halary S, Leigh JW, Cheaib B, Lopez P, Bapteste E (2010). Network analyses structure genetic diversity in independent genetic worlds. Proc. Natl. Acad. Sci..

[2] Jain R, Rivera MC, Moore JE, Lake JA (2003). Horizontal gene transfer accelerates genome innovation and evolution. Mol. Biol. Evol..

[3] Greve BO, Jensen S, Brügger K, Zillig W, Garrett RA (2004). Genomic comparison of archaeal conjugative plasmids from Sulfolobus. Archaea.

[4] Van Kranenburg R (2005). Functional analysis of three plasmids from *Lactobacillus**plantarum*. Appl. Environ. Microbiol..

[5] Sekine M (2006). Sequence analysis of three plasmids harboured in *Rhodococcus**erythropolis* strain PR4. Environ. Microbiol..

[6] Kuenne C (2010). Comparative analysis of plasmids in the genus *Listeria*. PLoS One.

[7] Dib JR (2013). Complete genome sequence of pAP13, a large linear plasmid of a *Brevibacterium* strain isolated from a saline lake at 4,200 meters above sea level in Argentina. Genome Announc..

[8] Dib JR (2013). First complete sequence of a giant linear plasmid from a *Micrococcus* strain isolated from an extremely high-altitude lake. Genome Announc..

[9] Dib JR (2015). Complete genome sequence of the linear plasmid pJD12 hosted by *Micrococcus* sp. D12, isolated from a high-altitude volcanic lake in Argentina. Genome Announc..

[10] Dib JR (2018). Complete genome sequences of pLMA1 and pLMA7, two large linear plasmids of *Micrococcus* strains isolated from a high-altitude lake in Argentina. Genome Announc..

[11] Salto IP (2018). Comparative genomic analysis of *Acinetobacter* spp. plasmids originating from clinical settings and environmental habitats. Sci. Rep..

[12] Dib JR, Liebl W, Wagenknecht M, Farías ME, Meinhardt F (2013). Extrachromosomal genetic elements in *Micrococcus*. Appl. Microbiol. Biotechnol..

[13] Zhang T, Zhang XX, Ye L (2011). Plasmid metagenome reveals high levels of antibiotic resistance genes and mobile genetic elements in activated sludge. PLoS One.

[14] Kav AB (2012). Insights into the bovine rumen plasmidome. Proc. Natl. Acad. Sci..

[15] Sentchilo V (2013). Community-wide plasmid gene mobilization and selection. ISME J..

[16] Jørgensen TS, Xu Z, Hansen MA, Sørensen SJ, Hansen LH (2014). Hundreds of circular novel plasmids and DNA elements identified in a rat cecum metamobilome. PLoS One.

[17] Kothari A (2019). Large circular plasmids from groundwater plasmidomes span multiple incompatibility groups and are enriched in multimetal resistance genes. MBio.

[18] Perez MF (2020). First report on the plasmidome from a high-altitude lake of the Andean Puna. Front. Microbiol..

[19] Dib JR, Wagenknecht M, Farías ME, Meinhardt F (2015). Strategies and approaches in plasmidome studies-uncovering plasmid diversity disregarding of linear elements?. Front. Microbiol..

[20] Farías M (2020). Microbial Ecosystems in Central Andes Extreme Environments: Biofilms, Microbial Mats, Microbialites and Endoevaporites.

[21] Vignale FA (2021). Lithifying and non-lithifying microbial ecosystems in the wetlands and salt flats of the Central Andes. Microb. Ecol..

[22] Saona Acuña LA, Soria MN, Villafañe PG, Stepanenko T, Farías ME, Farías M (2020). Arsenic and its biological role: From early Earth to current Andean microbial ecosystems. Microbial Ecosystems in Central Andes Extreme Environments.

[23] Ordoñez OF, Rasuk MC, Soria MN, Contreras M, Farías ME (2018). Haloarchaea from the Andean Puna: Biological role in the energy metabolism of arsenic. Microb. Ecol..

[24] Wagenknecht M, Pérez MF, Dib JR, Farias ME (2020). Linear megaplasmids spreading in the Andean resistome. Microbial Ecosystems in Central Andes Extreme Environments.

[25] Rascovan N, Maldonado J, Vazquez MP, Eugenia Farías M (2016). Metagenomic study of red biofilms from Diamante Lake reveals ancient arsenic bioenergetics in haloarchaea. ISME J..

[26] Saona LA (2019). Analysis of co-regulated abundance of genes associated with arsenic and phosphate metabolism in Andean Microbial Ecosystems. bioRxiv..

[27] Klindworth A (2013). Evaluation of general 16S ribosomal RNA gene PCR primers for classical and next-generation sequencing-based diversity studies. Nucleic Acids Res..

[28] Gantner S, Andersson AF, Alonso-Sáez L, Bertilsson S (2011). Novel primers for 16S rRNA-based archaeal community analyses in environmental samples. J. Microbiol. Methods.

[29] Bolger AM, Lohse M, Usadel B (2014). Trimmomatic: A flexible trimmer for Illumina sequence data. Bioinformatics.

[30] Bankevich A (2012). SPAdes: A new genome assembly algorithm and its applications to single-cell sequencing. J. Comput. Biol..

[31] Rozov R (2016). Recycler: An algorithm for detecting plasmids from de novo assembly graphs. Bioinformatics.

[32] Luo H, Gao F (2019). DoriC 10.0: An updated database of replication origins in prokaryotic genomes including chromosomes and plasmids. Nucleic Acids Res..

[33] Langmead B, Salzberg SL (2012). Fast gapped-read alignment with Bowtie 2. Nat. Methods.

[34] Seemann T (2014). Prokka: Rapid prokaryotic genome annotation. Bioinformatics.

[35] Meyer F (2008). The metagenomics RAST server—A public resource for the automatic phylogenetic and functional analysis of metagenomes. BMC Bioinform..

[36] Parks DH, Beiko RG (2010). Identifying biologically relevant differences between metagenomic communities. Bioinformatics.

[37] Xie Y (2018). TADB 2.0: An updated database of bacterial type II toxin–antitoxin loci. Nucleic Acids Res..

[38] Leplae R, Lima-Mendez G, Toussaint A (2010). ACLAME: A CLAssification of Mobile genetic Elements, update 2010. Nucleic Acids Res..

[39] Pal C, Bengtsson-Palme J, Rensing C, Kristiansson E, Larsson DGJ (2014). BacMet: Antibacterial biocide and metal resistance genes database. Nucleic Acids Res..

[40] Chen L (2005). VFDB: A reference database for bacterial virulence factors. Nucleic Acids Res..

[41] Altschul SF (1997). Gapped BLAST and PSI-BLAST: A new generation of protein database search programs. Nucleic Acids Res..

[42] Li W, Jaroszewski L, Godzik A (2002). Sequence clustering strategies improve remote homology recognitions while reducing search times. Protein Eng..

[43] Sievers F (2011). Fast, scalable generation of high-quality protein multiple sequence alignments using Clustal Omega. Mol. Syst. Biol..

[44] Mistry J, Finn RD, Eddy SR, Bateman A, Punta M (2013). Challenges in homology search: HMMER3 and convergent evolution of coiled-coil regions. Nucleic Acids Res..

[45] Jia B (2017). CARD 2017: Expansion and model-centric curation of the comprehensive antibiotic resistance database. Nucleic Acids Res..

[46] Xie Z, Tang H (2017). ISEScan: Automated identification of insertion sequence elements in prokaryotic genomes. Bioinformatics.

[47] Riadi G, Medina-Moenne C, Holmes DS (2012). TnpPred: A web service for the robust prediction of prokaryotic transposases. Comp. Funct. Genomics.

[48] Caporaso JG (2010). QIIME allows analysis of high-throughput community sequencing data. Nat. Publ. Gr..

[49] Zhang J, Kobert K, Flouri T, Stamatakis A (2014). PEAR: A fast and accurate Illumina Paired-End reAd mergeR. Bioinformatics.

[50] Martin M (2011). Cutadapt removes adapter sequences from high-throughput sequencing reads. EMBnet.journal.

[51] Edgar RC (2010). Search and clustering orders of magnitude faster than BLAST. Bioinformatics.

[52] Yilmaz P (2014). The SILVA and ‘all-species Living Tree Project (LTP)’ taxonomic frameworks. Nucleic Acids Res..

[53] Makarova KS, Wolf YI, Koonin EV (2019). Towards functional characterization of archaeal genomic dark matter. Biochem. Soc. Trans..

[54] Albarracín VH (2012). Extremophilic *Acinetobacter* strains from high-altitude lakes in Argentinean Puna: Remarkable UV-B resistance and efficient DNA damage repair. Orig. Life Evol. Biosph..

[55] Fernández Zenoff V, Siñeriz F, Farías ME (2006). Diverse responses to UV-B radiation and repair mechanisms of bacteria isolated from high-altitude aquatic environments. Appl. Environ. Microbiol..

[56] Flores MR, Ordoñez OF, Maldonado MJ, Farías ME (2009). Isolation of UV-B resistant bacteria from two high altitude Andean lakes (4,400 m) with saline and non saline conditions. J. Gen. Appl. Microbiol..

[57] Ordoñez OF, Flores MR, Dib JR, Paz A, Farías ME (2009). Extremophile culture collection from Andean lakes: Extreme pristine environments that host a wide diversity of microorganisms with tolerance to UV radiation. Microb. Ecol..

[58] Kurth D (2015). Genomic and proteomic evidences unravel the UV-resistome of the poly-extremophile *Acinetobacter* sp. Ver3. Front. Microbiol..

[59] Sancho-Tomás M (2020). Geochemical evidence for arsenic cycling in living microbialites of a high altitude Andean lake (Laguna Diamante, Argentina). Chem. Geol..

[60] Li LL, Norman A, Hansen LH, Sørensen SJ (2012). Metamobilomics—Expanding our knowledge on the pool of plasmid encoded traits in natural environments using high-throughput sequencing. Clin. Microbiol. Infect..

[61] Bateman A, Coggill P, Finn RD (2010). DUFs: Families in search of function. Acta Crystallogr. Sect. F Struct. Biol. Cryst. Commun..

[62] El-Gebali S (2019). The Pfam protein families database in 2019. Nucleic Acids Res..

[63] Schlüter A, Krause L, Szczepanowski R, Goesmann A, Pühler A (2008). Genetic diversity and composition of a plasmid metagenome from a wastewater treatment plant. J. Biotechnol..

[64] Shi Y, Zhang H, Tian Z, Yang M, Zhang Y (2018). Characteristics of ARG-carrying plasmidome in the cultivable microbial community from wastewater treatment system under high oxytetracycline concentration. Appl. Microbiol. Biotechnol..

[65] Garcillán-Barcia MP, Francia MV, De La Cruz F (2009). The diversity of conjugative relaxases and its application in plasmid classification. FEMS Microbiol. Rev..

[66] Garcillán-Barcia MP, Redondo-Salvo S, Vielva L, de la Cruz F (2020). MOBscan: Automated annotation of MOB relaxases. Methods Mol. Biol..

[67] Arcus VL, McKenzie JL, Robson J, Cook GM (2011). The PIN-domain ribonucleases and the prokaryotic VapBC toxin–antitoxin array. Protein Eng. Des. Sel..

[68] Bolhuis H, Te Poele EM, Rodriguez-Valera F (2004). Isolation and cultivation of Walsby’s square archaeon. Environ. Microbiol..

[69] Page R, Peti W (2016). Toxin–antitoxin systems in bacterial growth arrest and persistence. Nat. Chem. Biol..

[70] Khelaifia S, Drancourt M (2012). Susceptibility of archaea to antimicrobial agents: Applications to clinical microbiology. Clin. Microbiol. Infect..

[71] Cavicchioli R, Curmi PMG, Saunders N, Thomas T (2003). Pathogenic archaea: Do they exist?. BioEssays.

[72] Lepp PW (2004). Methanogenic Archaea and human periodontal disease. Proc. Natl. Acad. Sci. U.S.A..

[73] Andres J, Bertin PN (2016). The microbial genomics of arsenic. FEMS Microbiol. Rev..

[74] Ben Fekih I (2018). Distribution of arsenic resistance genes in prokaryotes. Front. Microbiol..

[75] Wang G, Kennedy SP, Fasiludeen S, Rensing C, DasSarma S (2004). Arsenic resistance in *Halobacterium* sp. strain NRC-1 examined by using an improved gene knockout system. J. Bacteriol..

[76] Werner J (2014). *Halorhabdus tiamatea*: Proteogenomics and glycosidase activity measurements identify the first cultivated euryarchaeon from a deep-sea anoxic brine lake as potential polysaccharide degrader. Environ. Microbiol..

[77] Filée J, Siguier P, Chandler M (2007). Insertion sequence diversity in archaea. Microbiol. Mol. Biol. Rev..

[78] Craig, N. *Mobile DNA III* (2020).

[79] Szuplewska M, Czarnecki J, Bartosik D (2015). Autonomous and non-autonomous Tn 3-family transposons and their role in the evolution of mobile genetic elements. Mob. Genet. Elem..

[80] Frost LS, Leplae R, Summers AO, Toussaint A (2005). Mobile genetic elements: The agents of open source evolution. Nat. Rev. Microbiol..

[81] Hülter N (2017). An evolutionary perspective on plasmid lifestyle modes. Curr. Opin. Microbiol..

[82] Ng WV (2000). Genome sequence of *Halobacterium* species NRC-1. Proc. Natl. Acad. Sci. U.S.A..

[83] Baliga NS (2004). Genome sequence of *Haloarcula**marismortui*: A halophilic archaeon from the Dead Sea. Genome Res..

[84] Pfeiffer F (2008). Evolution in the laboratory: The genome of *Halobacterium**salinarum* strain R1 compared to that of strain NRC-1. Genomics.

[85] Saunders E (2010). Complete genome sequence of *Haloterrigena**turkmenica* type strain (4k T). Stand. Genomic Sci..

[86] Hartman AL (2010). The complete genome sequence of *Haloferax**volcanii* DS2, a model Archaeon. PLoS One.

[87] Roh SW (2010). Complete genome sequence of *Halalkalicoccus jeotgali* B3T, an extremely halophilic archaeon. J. Bacteriol..

[88] Malfatti S (2009). Complete genome sequence of *Halogeometricum borinquense* type strain (PR3T). Stand. Genomic Sci..

[89] Anderson I (2012). Complete genome sequence of *Halopiger xanaduensis* type strain (SH-6 T). Stand. Genomic Sci..

[90] Ding JY, Chiang PW, Hong MJ, Dyall-Smith M, Tang SL (2014). Complete genome sequence of the extremely halophilic archaeon *Haloarcula**hispanica* strain N601. Genome Announc..

[91] Castillo AM (2006). *Halostagnicola**larsenii* gen. nov., sp. Nov., an extremely halophilic archaeon from a saline lake in Inner Mongolia, China. Int. J. Syst. Evol. Microbiol..

