# Supplementary material for: Assessment of the plasmidome of an extremophilic microbial community from the Diamante Lake, Argentina
Source: Sci Rep. 2021 Nov 2;11:21459. doi: 10.1038/s41598-021-00753-1 (PMC8563766; doi:10.1038/s41598-021-00753-1)
Supplement: Supplementary file 1 — Supplementary Figures. [file 41598_2021_753_MOESM1_ESM.pdf]

Assessment of the plasmidome of an extremophilic microbial community from the Diamante Lake, Argentina

María Florencia Perez, Luis Alberto Saona, María Eugenia Farías, Anja Poehlein, Friedhelm Meinhardt, Rolf Daniel, Julián Rafael Dib

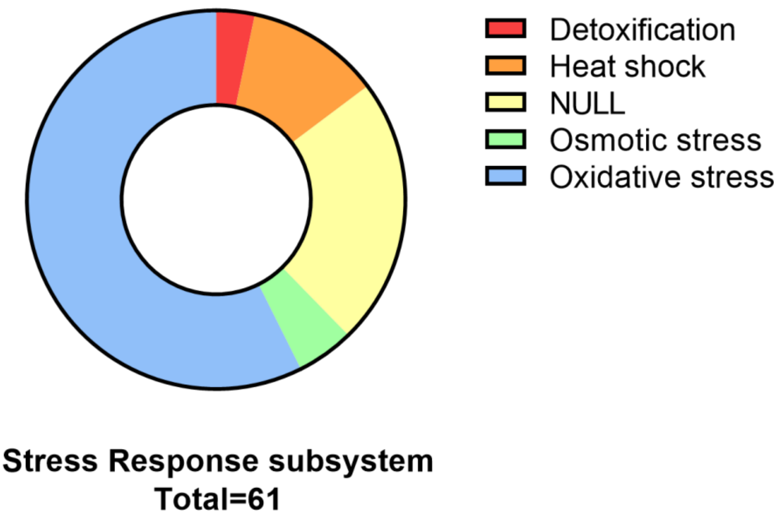

Supplementary Figure S1. Predicted functional profile of the Diamante Lake plasmidome. "Stress Response" subsystem level 2 classification of the SEED database (*E*-value $\leq 10^{-5}$).

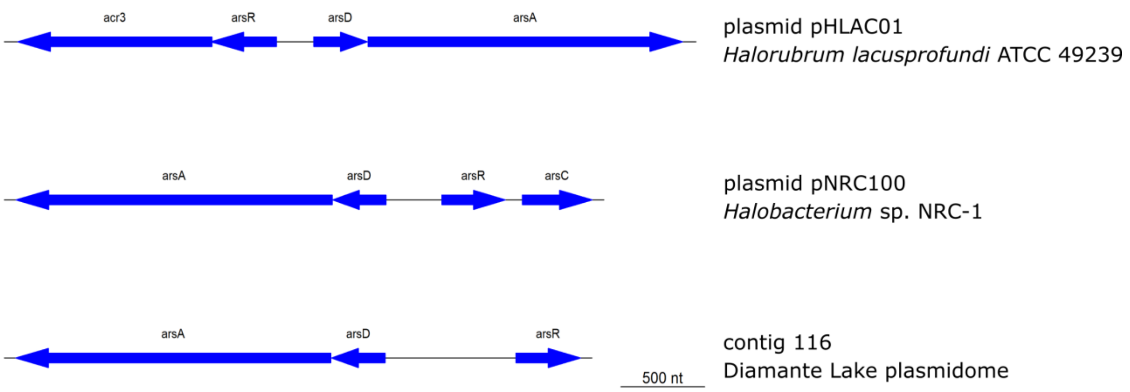

Supplementary Figure S2. Genetic organization of the *ars* operons in the contig 116 of the Diamante Lake plasmidome and in plasmids of haloarchaeal strains. The gene maps were created using the R package genoplotR[1].

Reference

1 Guy, L., Roat Kultima, J. & Andersson, S. G. E. genoPlotR: comparative gene and genome visualization in R. *Bioinformatics* **26**, 2334–2335 (2010).

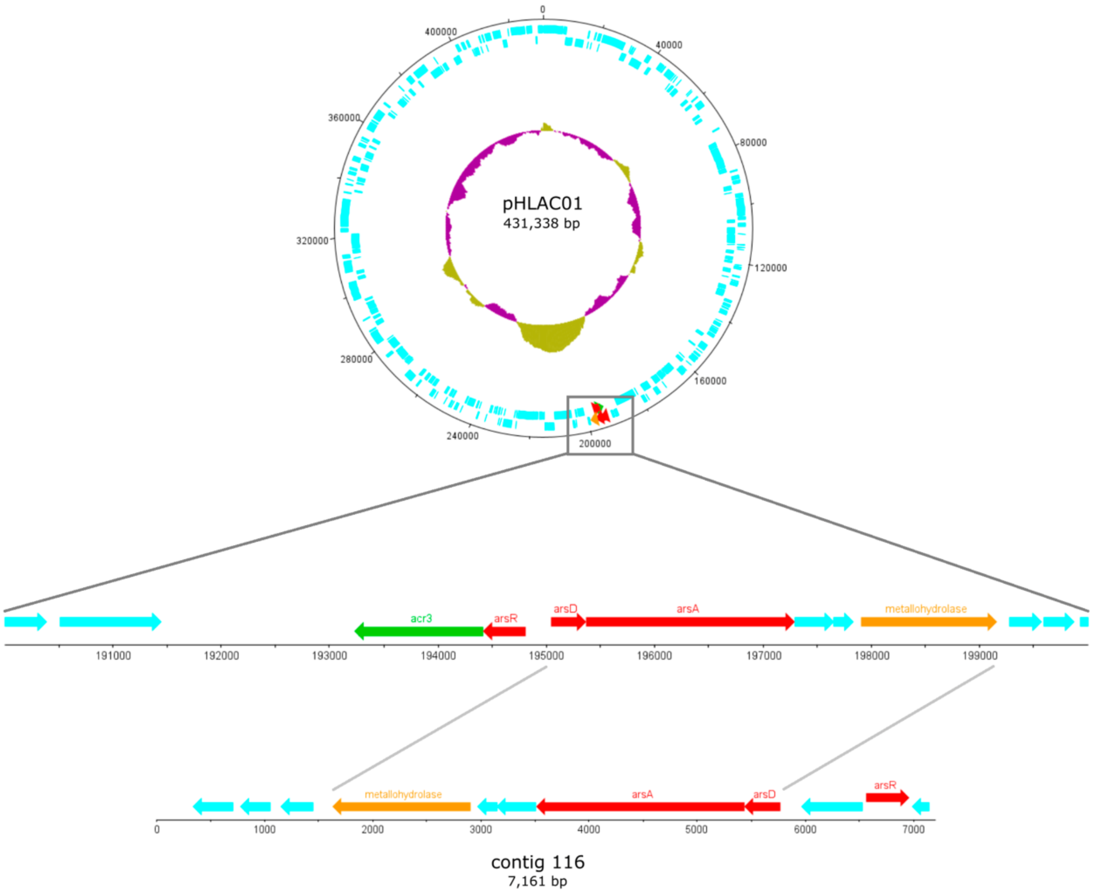

Supplementary Figure S3. Matches between plasmid pHLAC01 of *Halorubrum lacusprofundi* ATCC 49239 and contig 116 of the Diamante Lake plasmidome. Maps were generated using DNAplotter[2].

Reference

2. Carver, T., Thomson, N., Bleasby, A., Berriman, M. & Parkhill, J. DNAPlotter: circular and linear interactive genome visualization. *Bioinformatics* **25**, 119–120 (2009).
